# Impact of Multiple Climate Stressors on Early Life Stages of North Pacific Kelp Species

**DOI:** 10.1002/ece3.71661

**Published:** 2025-06-20

**Authors:** Veronica Farrugia Drakard, Jordan A. Hollarsmith, Michael S. Stekoll

**Affiliations:** ^1^ Department of Botany Faculty of Science, University of British Columbia Vancouver British Columbia Canada; ^2^ Alaska Fisheries Science Center, National Marine Fisheries Service National Oceanic and Atmospheric Administration Juneau Alaska USA; ^3^ College of Fisheries and Ocean Sciences, University of Alaska Fairbanks Juneau Alaska USA

## Abstract

This study examines the effects on bull kelp (*Nereocystis luetkeana*) and ribbon kelp ( *Alaria marginata* ) of combinations of three climate‐related stressors relevant to high‐latitude kelp forests: temperature, salinity, and sediment load. Fertile specimens of both species were collected from Juneau, Alaska. Spores produced were cultivated over 40 days in four ecologically relevant stressor treatments: control (all stressor levels normal; CTRL), increased glacial melt (normal temperature, low salinity, high sediment load; GLAC), increased runoff (high temperature, low salinity, normal sediment load; MELT) and climate change (high temperature, low salinity, high sediment load; CLIM). Gametophyte density in both species was reduced in treatments involving high sediment load. Gametophyte density in bull kelp was also reduced in the increased runoff treatment, while ribbon kelp appeared resilient. Gametophytes of *A. marginata* grew equally in the increased glacial melt treatment as in the control and exhibited some growth in the increased runoff treatment. Conversely, gametophytes of *N. luetkeana* exhibited low growth in all treatments other than the control. A large number of gametophytes of both species were unidentifiable as either male or female in high‐temperature treatments. This likely had impacts on reproduction, as neither species was able to produce eggs or sporophytes in these treatments. The results presented here show that both *N. luetkeana* (a subtidal canopy‐former) and *A. marginata* (an intertidal subcanopy species) are sensitive to combinations of thermal, hyposaline, and sediment stress. This may have an impact on the development of gametophytes and successful reproduction in these species and may therefore have implications for the ongoing persistence of wild kelp populations in future ocean conditions.

## Graphical abstract

In this study, we examine the impacts of four ecologically relevant stressor treatments on spore germination, gametophyte density, gametophyte growth, and reproductive development in the aforementioned species. Our results reveal significant negative impacts of sediment deposition on gametophyte density and of high temperature on reproductive development. Additionally, we discuss species‐level variation with a view to the ecological niches of ribbon and bull kelp. These results are presented in the context of ongoing climate change and glacial melt, and we offer an assessment of how these impacts on early life‐stages translate to effects on wild kelp populations.

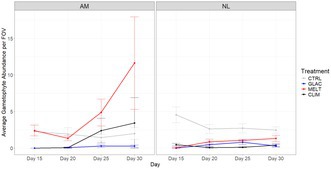

## 1 Introduction

As climate change progresses, the marine environment is being altered in complex and often unpredictable ways. This has already resulted in impacts on a wide range of organisms, including shifts in species distributions and local declines in abundance. Kelp forests are no exception: California reported a 90% loss of bull kelp canopy cover between 2014 and 2017, resulting in dramatic changes in associated biota and the collapse of several fisheries in the area (Korabik et al. 2023; Rogers‐Bennett and Catton 2019). In the North Atlantic, several independent studies from the Iberian Peninsula have reported declines in abundance and range contractions for *Laminaria hyperborea, L*. *ochroleuca* , and *Saccharina latissima* (see Smale 2019). Global climate change is associated with a number of climate‐related stressors that are likely to be contributing to these impacts, including a rise in sea surface temperature, changes in nutrient supply, hyposaline stress and increased sediment deposition, ongoing ocean acidification and changes in light availability and quality in the water column (see Farrugia Drakard et al. 2023).

Of these, the stressors most likely to have a significant impact on high‐latitude kelp forests in the coming decades are temperature, hyposaline stress, and sedimentation. The majority of studies involving kelp species have thus far focused on temperature (Farrugia Drakard et al. 2023). This tends to reflect climate priorities, as a rise in sea surface temperature and an increase in the frequency of marine heatwaves are both likely to result in major climate‐related impacts on marine systems over the coming decades (Smale 2019; Smale et al. 2019). Rising temperatures have resulted in shifting species distributions, including range contractions for cold‐temperate species and range expansions for warm‐adapted species (Goldsmit et al. 2021; Wilson et al. 2019). The Arctic is warming approximately four times faster than the rest of the globe due to polar amplification (Rantanen et al. 2022) and therefore high‐latitude kelp species are likely to be particularly vulnerable in a warming climate.

Additionally, regions at high latitudes will be subject to increased glacial melt as climate change progresses. This contributes to hyposaline conditions and increased sediment deposition into the marine environment at glacial outflows. Both hyposaline stress and sediment load have been shown to impact kelp physiology and the provision of ecosystem services (Picard et al. 2022; Deiman et al. 2012; Farrugia Drakard et al. 2025). Hyposaline stress has been associated with reduced photosynthetic capacity and a loss of photosynthetic pigments (Karsten 2007; Li et al. 2020; Monteiro et al. 2019; Spurkland and Iken 2011). Studies have also noted declines in sporophyte and gametophyte growth rates and spore settlement densities (Buschmann et al. 2004; Lind and Konar 2017; Monteiro et al. 2021; Muth et al. 2021). Light attenuation due to increased sediment load in the water column has been shown to impact gametophyte growth and sporophyte production during recruitment windows (Shaffer and Parks 1994; Zacher et al. 2016). Additionally, increases in mortality due to sediment scour have consequences for overall species densities (Dean and Deysher 1983; Devinny and Voise 1978).

The impacts of climate stressors on kelp forests should be of great concern to us, as kelp form the basis of highly productive ecosystems, constitute the basis of inshore trophic networks, and provide habitat to numerous associated organisms (Smale et al. 2020; Teagle et al. 2017). Kelp are therefore considered to be ecosystem engineers (Arnold et al. 2017). Any change which impacts such a foundational organism is likely to have consequences for the ecosystem as a whole. Additionally, various species of kelp have commercial importance as food products, fodder for animals, and for the provision of chemical derivatives such as alginate and carrageenan (Stekoll and Else 1992). Kelp aquaculture is a highly successful commercial enterprise on a global scale and is rapidly expanding (Kim et al. 2019; Peteiro and Sánchez 2012; Stekoll et al. 2021).

Both ribbon kelp ( *Alaria marginata* ) and bull kelp (*Nereocystis luetkeana*) are of major ecological and commercial importance in the northeast Pacific. *N. luetkeana* is a subtidal canopy‐forming species which has suffered extensive declines along parts of its range in recent years, though populations appear to be stable in Alaska (Supratya and Martone 2023; Hollarsmith et al. 2020). *A. marginata* is an intertidal subcanopy species and is therefore exposed to frequent hyposaline stress and increased sediment load due to freshwater influx from rainfall and riverine input. Comparatively, bull kelp is exposed to these stressors only infrequently and likely only at the level of the surface canopy. Both of these species are most commonly annuals, though select individuals or populations may be perennial (pers. obsv.). Both species experience massive spore production in the late summer and early autumn, followed by the persistence of microscopic life‐stages (spores, gametophytes, and juvenile sporophytes) on the benthos until around late spring. Juvenile sporophytes undergo rapid maturation in mid‐to‐late spring, resulting in the persistence of populations from year to year.

While historically most studies have considered the impacts of climate stressors individually, there has been growing interest in utilizing multiple stressor studies. This reflects a growing awareness that the marine environment constitutes a system of interconnected components and influences, all of which will be affected in complex ways by a changing climate (Farrugia Drakard et al. 2023). Multiple stressor studies allow for an examination of stressor interactions. These include synergistic interactions (the combined effect of two stressors is greater than the added effects of each stressor individually), antagonistic interactions (the combined effect of two stressors is less than the added effects of each stressor individually), and additive interactions (the combined effect of two stressors is equivalent to the added effects of each stressor individually). In terms of studies involving kelp, most multiple stressor investigation have reported synergisms between stressors (Farrugia Drakard et al. 2023).

The majority of multiple stressor studies on kelp species have utilized two stressors varying simultaneously. Of these, the most commonly investigated stressor combinations are temperature and light, temperature and nutrients, or temperature and salinity (Farrugia Drakard et al. 2023). The present study is the first to utilize three climate‐related stressors identified as being the most impactful to high‐latitude kelp species: temperature, salinity, and sediment load. We identified environmentally realistic stressor levels based on an examination of species tolerances and climate projections, and combined these in a total of four ecologically relevant stressor treatments: a control (normal levels of all three stressors), a glacial runoff treatment (normal temperature, low salinity, high suspended sediment load), a meltwater runoff treatment (high temperature, low salinity, normal suspended sediment load), and a full‐stress climate change treatment (high temperature, low salinity, high suspended sediment load).

The aim of this study was to investigate the impact of these three stressors in combination on the survival and reproduction of early life‐stages of ribbon kelp ( *Alaria marginata* ) and bull kelp (*Nereocystis luetkeana*). We hypothesized that spore germination, gametophyte growth and development, and egg and sporophyte production of both species would be lower in all stressor treatments compared to the control. Additionally, we hypothesized that these response metrics would be lower in the full‐stress climate change treatment compared to both the glacial runoff treatment and the meltwater runoff treatment for both species considered.

## 2 Methodology

### 2.1 Sorus Collection and Sporulation

**FIGURE 1:**
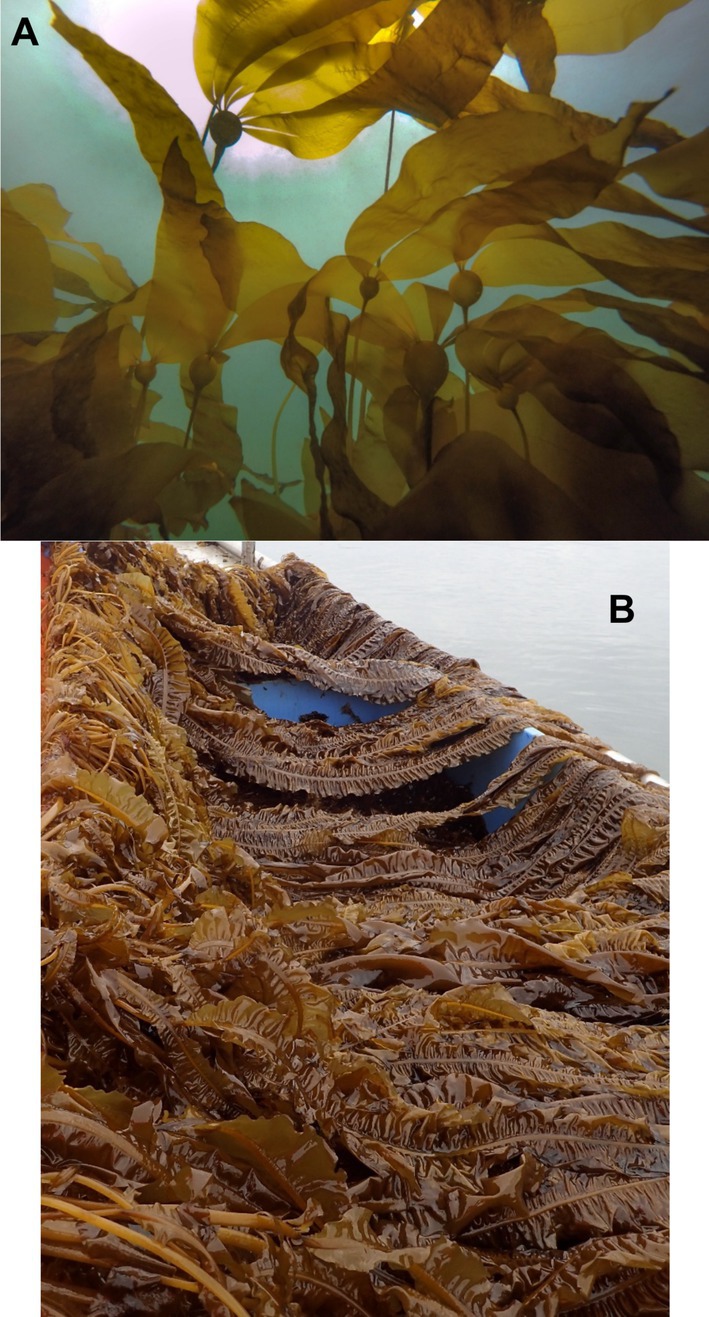
Images of *Nereocystis luetkeana* (bull kelp, A) and *Alaria marginata* (ribbon kelp, B). Photo credits: Tamsen Peeples.

**FIGURE 2:**
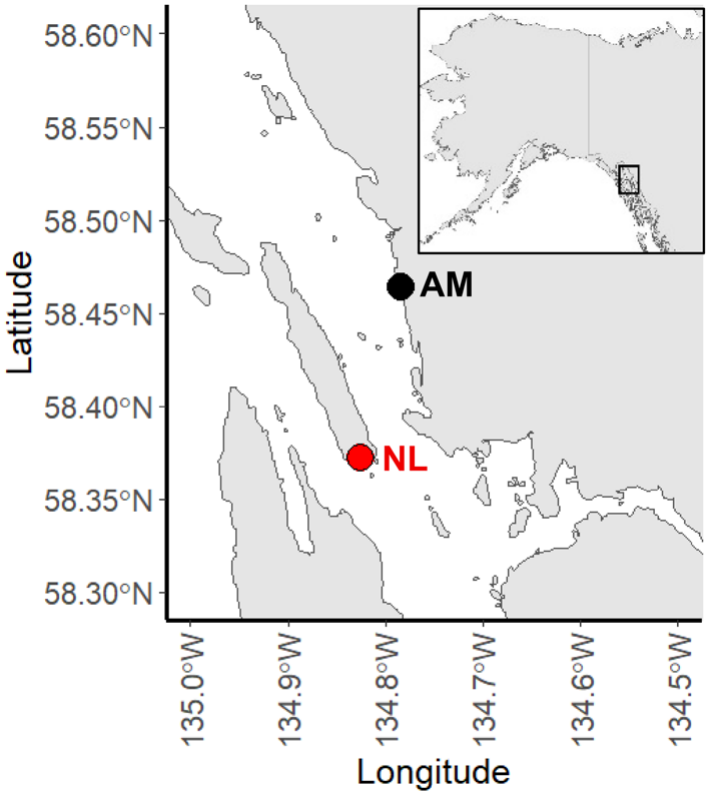
Map showing the study area and collection sites within Juneau, AK. *Alaria marginata* was collected from Site AM and *Nereocystis luetkeana* was collected from Site NL.

We collected fertile specimens of *Alaria marginata* and *Nereocystis luetkeana* (Figure 1) from Juneau, Alaska, in July 2024 (Figure 2). This area experiences significant glacial influence from the glaciers of the Juneau Icefield (Ziemen et al. 2016). The most significant glacier in terms of this experiment is the Mendenhall Glacier, which terminates in Mendenhall Lake and discharges into expansive estuarine wetlands (Siegela 1988). This region is therefore subject to freshwater input from glacial melt, rainfall, and snowmelt. Kelp populations established here are likely to experience significant impacts related to rising temperatures and glacial melt over the coming decades.

Sporophylls from 10 individuals of each species were cleaned in 10% (v/v) iodine solution (Betadine) in freshwater, dried with paper towels, and stored for 24 h in a cold (4°C), dark, dry environment. Sporophylls of each species were then separately placed in filtered, UV‐sterilized seawater at 12°C under fluorescent lighting (40–60 μmol m^−2^ s^−1^) for 1 h to induce sporulation. We filtered the resultant spore solutions through a 46 μm sieve and determined zoospore densities using a hemocytometer with an Improved Neubauer grid. Subsequently, the spore solutions were diluted with UV‐sterilized seawater to a density of 2000 zoospores/mL^−1^. This spore density was selected through multiple trials as being optimal for settlement and growth of gametophytes without crowding.

### 2.2 Experimental Set‐Up and Design

Two treatment levels were selected for each individual stressor, representing a “normal” condition and a “stressful” condition. These were defined as follows, with justification below:

- Temperature: T (normal) – 11°C, T+ (stressful) – 14°C.
- Salinity: S (normal) – 32, S− (stressful) – 15.
- Sediment: D (normal) – 0 g/L, D+ (stressful) – 0.1 g/L.

A temperature of 11°C is utilized in most hatchery operations and is considered the optimal growing temperature for the early life stages of both *A. marginata* and *N. luetkeana*, while 14°C was the maximum recorded SST in Juneau between 2009 and 2012 (data from Station JNEA2, citation: National Oceanic and Atmospheric Administration 2012). For the purposes of this experiment, 14°C is also considered to represent a realistic future climate scenario based on the Representative Concentration Pathways (RCPs) formally adopted by the Intergovernmental Panel on Climate Change (IPCC 2019). These are a set of climate change scenarios used to project future emissions and their impacts. This experiment uses RCP 6.0 as a reference, which predicts stabilization of total radiative forcing by 2100. A salinity of 32 is fully oceanic, while 15 is the approximate lower tolerance threshold for Arctic kelp species (Karsten 2007). A sediment level of 0 g/L is the most likely scenario for kelp in oceanic conditions, while 0.1 g/L is a realistic scenario for kelp beds exposed to glacial outflow in Juneau, based on turbidity measurements taken along the Juneau coastline every 2 weeks between September 2023 and March 2024 (Farrugia Drakard et al., under review).

Of the eight possible treatment combinations arising from these stressor levels, four experimental treatments were selected as being representative of potential real‐world ecological conditions. These were defined as follows:

- T/S/D—all levels normal, control treatment (CTRL).
- T/S−/D+ − temperature normal, low salinity, and high sediment, glacial runoff treatment (GLAC).
- T+/S−/D− sediment load normal, high temperature, and low salinity, meltwater treatment (MELT).
- T+/S−/D+ − all levels stressful, climate change treatment (CLIM).

For each treatment combination, we filled 5 petri dishes of surface area 23.76 cm^2^ (5.5 cm diameter) with 15 mL of the 2000 zoospores/mL^−1^ spore solution (12.63 spores/mm^2^), for a total of 20 petri dishes per species and 40 petri dishes for the whole experiment. These were stored in the dark at 12°C for 48 h to allow zoospore settlement. After 48 h, the petri dishes were rinsed with seawater to remove non‐vital spores, and microphotographs were taken at 200× phase contrast magnification of 5 haphazardly selected fields of view per petri dish using a Leica DMi8 S inverted microscope. These microphotographs were used to determine the average initial settled zoospore count.

Separate culture media were prepared for the two salinity levels utilized in this experiment as follows: for 1000 mL of (a) 32–988 mL UV‐sterilized seawater, 10 mL Provasoli's enriched seawater medium with iodine (PESI) working solution (Provasoli 1968; Tatewaki 1966), 2 mL 0.25 g/L GeO_2_ solution, and (b) 15–458 mL UV‐sterilized seawater, 530 mL Milli‐Q water, 10 mL PESI working solution, 2 mL 0.25 g/L GeO_2_ solution. Salinities were checked after preparation of the culture media using a handheld refractometer. The nutrient concentrations utilized in this experiment were taken from the New England Seaweed Culture Handbook (Redmond et al. 2014) and are considered to be standard and optimum for kelp culturing.

Samples of glacial silt were collected from two locations along the Mendenhall River. These were dried and sterilized by heating to 70°C, allowing the silt to cool to room temperature and subsequently re‐heating to 70°C. To prepare culture media for the D+ treatments, 0.1 g/L of dry, sterilized glacial silt were added to the culture media prepared as described above. All D+ culture media were thoroughly mixed prior to use to ensure even distribution of the suspended sediment.

The solution in each petri dish was decanted out and replaced with 15 mL of the appropriate culture medium—5 petri dishes per treatment combination for each species. We then placed the petri dishes on shaking tables at 90 rotations/min in Percival Scientific incubators set at either 11°C (T) or 14°C (T+), light intensity 40–60 μmol m^−2^ s,^−1^ and a L:D regime of 12 h:12 h. This level of rotation was sufficient to keep most of the sediment suspended at all times. Microphotographs at 200× magnification of 5 haphazardly chosen fields of view per petri dish were taken after 5 and 10 days. These were used to determine the average number of zoospores and average number of germinated zoospores per treatment combination. Subsequently, microphotographs of 10 haphazardly chosen gametophytes per petri dish were taken at 15, 20, 25, 35, and 40 days. These were used to determine the average gametophyte size, gametophyte sex ratios, and the average number of eggs and sporophytes produced per female for each treatment combination. Microphotographs at 50× magnification of 5 fields of view per petri dish were also taken at these timepoints. All gametophytes in these microphotographs were counted to determine the average gametophyte density per field of view for every treatment combination. Medium changes were conducted every 5 days until the conclusion of the experiment after 40 days.

### 2.3 Data Analysis

All statistical analyses were carried out using R version 4.0.2 in RStudio version 2024.04.2 Build 764 (R Core Team 2021).

For each sampling point up to 10 days, the average number of zoospores and the average number of germinated zoospores per petri dish (*N* = 5 fields of view) were used to calculate the average proportion of zoospores germinated for each species and each treatment combination (*N* = 5 petri dishes). These data were arcsine‐transformed and analyzed using a three‐way mixed ANOVA with Time (0, 5, 10DAY) as a within‐subjects factor and Species (AM vs. NL) and Treatment (CTRL, GLAC, MELT, CLIM) as between‐subjects factors. *Post hoc* pairwise ANOVAs with Bonferroni correction were performed to explore significant effects.

For sampling points between 15 and 40 days, the average gametophyte size per species and treatment combination (*N* = 5 petri dishes) was calculated as the average of the lengths of each photographed gametophyte measured along its longest axis (*N* = up to 10 gametophytes). Both male and female gametophytes were included. These data were square root transformed and analyzed using a three‐way ANOVA with Time (15, 20, 25, 30, 25, 40DAY) as a within‐subjects factor and Species (AM vs. NL) and Treatment (CTRL, GLAC, MELT, CLIM) as between‐subjects factors. *Post hoc* pairwise ANOVAs with Bonferroni correction were performed to explore significant effects.

For sampling points between 15 and 30 days, the average gametophyte abundance per species and treatment combination (*N* = 5 petri dishes) was calculated as the average of the number of gametophytes observed per petri dish (*N* = 5 fields of view) for each species/treatment combination. These data were log transformed. Additionally, the average numbers of eggs and sporophytes produced per female were calculated for sampling points from 15 to 40 days and used to obtain averages per species and treatment combination. These data were square‐root transformed. Both these datasets were analyzed using three‐way mixed ANOVAs as described for gametophyte length above.

Finally, the average number of male, female, and unidentified gametophytes per petri dish were calculated from 15 days onwards and used to calculate sex ratios per species and treatment combination. Data for the number of identified and number of unidentified gametophytes were analyzed using three‐way mixed ANOVAs as described above.

## 3 Results

### 3.1 Zoospore Survival and Germination

**FIGURE 3:**
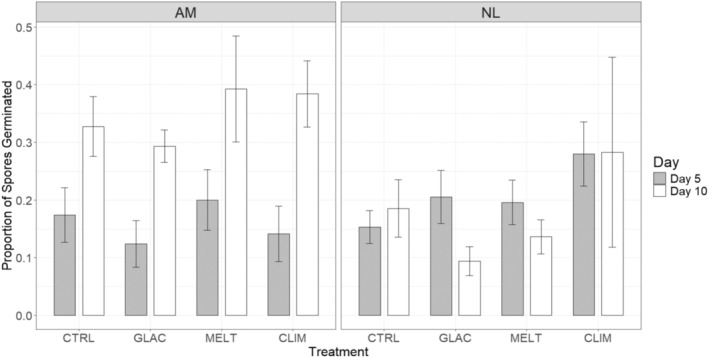
Mean proportion of *Alaria marginata* (AM) and *Nereocystis luetkeana* (NL) zoospores germinated at Day 5 and Day 10 across stressor treatments. Treatments are control (CTRL), climate change (CLIM), glacial runoff (GLAC) and meltwater (MELT), *n* = 5 petri dishes per treatment and species combination.

Overall, there appeared to be no effect of treatment combination on the proportion of spores germinated in either species (Figure 3 and Table S1). Spores of *Alaria marginata* germinated more successfully regardless of treatment, and this difference was significant (Figure 3 and Table S1). The proportion of *A. marginata* spores germinated increased from Day 5 to Day 10 across treatments, while the proportion of *Nereocystis luetkeana* spores germinated decreased or remained roughly the same (Figure 3). We observed a significant two‐way interaction between Species and Time (*F*_*1,32*_ = 9.318, *p* < 0.05; Table S1), confirming that there was a significant difference in the proportion of spores germinated between the two species, and this difference varied with time.

### 3.2 Gametophyte Density

**FIGURE 4:**
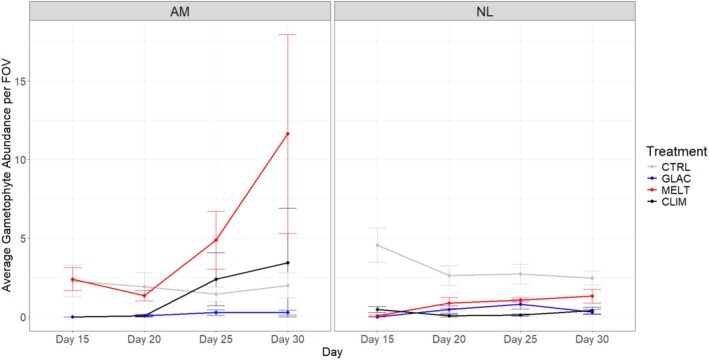
Mean gametophyte density per FOV (field of view) for *Alaria marginata* (AM) and *Nereocystis luetkeana* (NL) from Day 15 to Day 30 across stressor treatments. Error bars showing standard error. Treatments are control (CTRL), climate change (CLIM), glacial runoff (GLAC) and meltwater (MELT), *n* = 5 petri dishes per treatment and species combination.

Conversely, treatment did have an effect on gametophyte density. Density increased with time across species and treatments (main effect of Time: *F*_1.59,49.27_ = 4.254, *p* < 0.05; Figure 4 and Table S2). However, there was a difference in the number of gametophytes counted between treatments, and this difference varied between the two species (two‐way interaction Species × Treatment: *F*_3,31_ = 5.321, *p* < 0.05).

The effect of Treatment was significant for each species (Table S3). For *A. marginata*, gametophyte density was higher in the MELT treatment, lower in the CLIM and CTRL treatments, and lowest in the GLAC treatment (Figure 3; pairwise comparisons in Table S4). For *N. luetkeana*, gametophyte density was highest in the CTRL treatment, lower in the MELT treatment, and lowest in the CLIM and GLAC treatments (Figure 4; pairwise comparisons in Table S4).

### 3.3 Gametophyte Size and Sex Ratios

**FIGURE 5:**
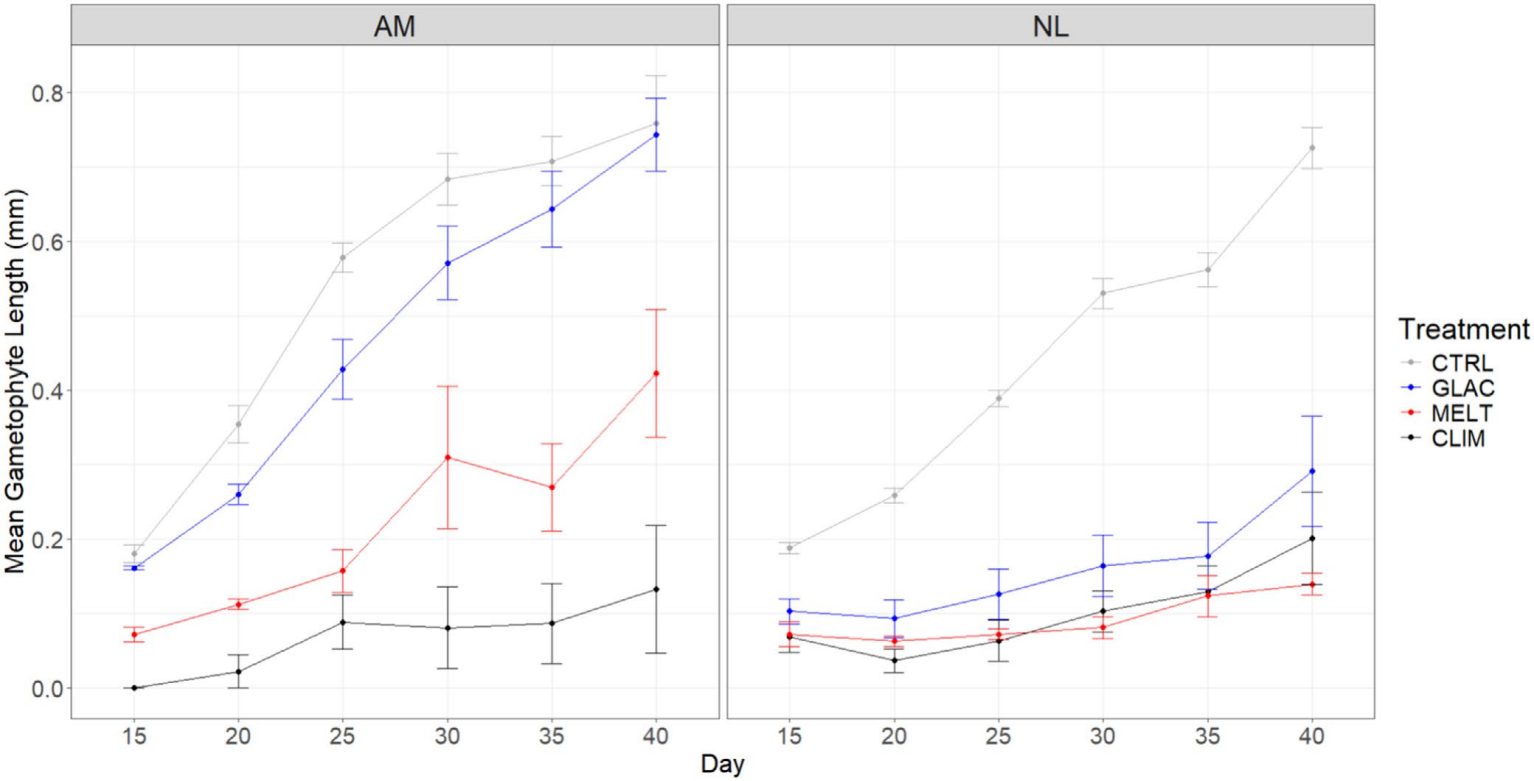
Mean gametophyte length for *Alaria marginata* (AM) and *Nereocystis luetkeana* (NL) from Day 15 to Day 40 across stressor treatments. Error bars show standard error. Treatments are control (CTRL), climate change (CLIM), glacial runoff (GLAC) and meltwater (MELT), *n* = 5 petri dishes per treatment and species combination.

In terms of gametophyte size, we found significant two‐way interactions between Treatment and Time (*F*_8.88,94.68_ = 2.112, *p* < 0.05), between Species and Time (*F*_2.96,94.68_ = 5.803, *p* < 0.05), and between Species and Treatment (*F*_3,32_ = 9.116, *p* < 0.05; Table S5). *Alaria marginata* appeared to exhibit some degree of resilience to high‐stress combinations: gametophyte length for this species was only slightly lower in the GLAC treatment than in the CTRL treatment at all timepoints, and some growth was still evident in the MELT treatment (Figure 5). Comparatively, *Nereocystis luetkeana* gametophytes exhibited lowered growth in all treatment combinations compared to the CTRL across all timepoints (Figure 5).

In terms of the interaction between Species and Treatment, we observed differences between species in the GLAC and MELT treatments (Table S6). Overall, both *A. marginata* and *N. luetkeana* grew best in the CTRL treatment, and both did poorly in the CLIM treatment (Figure 5). We also observed differences between treatments for both species (Table S6). Overall, there were significant differences between all pairs of treatments for *A. marginata* , while in *N. luetkeana* the CTRL treatment was significantly different from all other treatments (pairwise comparisons in Table S7).

**FIGURE 6:**
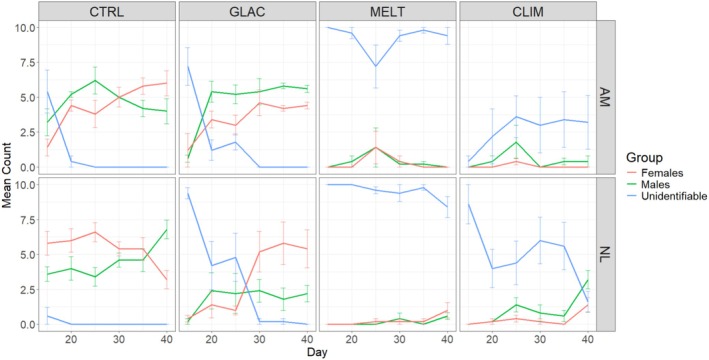
Mean number of male, female, and unidentified gametophytes per petri dish for *Alaria marginata* (AM) and *Nereocystis luetkeana* (NL) cultures from Day 15 to Day 40 across stressor treatments. Error bars show standard error. Treatments are control (CTRL), climate change (CLIM), glacial runoff (GLAC) and meltwater (MELT), *n* = 5 petri dishes per treatment and species combination.

For the most part, approximately equal numbers of male and female gametophytes were observed for both species across treatments at all timepoints (Figure 6). There were a few notable exceptions. In particular, we observed far fewer males than females for *N. luetkeana* in the GLAC treatment from Day 30 onwards, and far fewer males for *N. luetkeana* than for *A. marginata* in the same treatment at the same timepoints (Figure 6). Additionally, a significant number of gametophytes were severely deformed and unidentifiable as either male or female for both species in the high temperature treatments—CLIM and MELT (Figure 6 and Figures S1 and S2).

The number of gametophytes of unidentifiable sex was subject to a significant three‐way Species × Treatment × Time interaction (*F*_9.64,102.86_ = 3.386, *p* < 0.05; Table S8). Upon splitting the dataset by species, a two‐way Treatment × Time interaction was significant for both *A. marginata* and *N. luetkeana* (Table S9). The number of gametophytes of unidentifiable sex dropped with time in both the CTRL and GLAC treatments for both species but remained relatively high and constant in the MELT treatment for both species and the CLIM treatment for *A. marginata* (Figure 6). For *N. luetkeana* in the MELT treatment, the number of gametophytes of unidentifiable sex dropped at 40 days (Figure 6; pairwise comparisons in Table S10). Overall, the number of gametophytes of unidentifiable sex was highest in the MELT treatment, lower in the CLIM treatment, and lowest in the CTRL and GLAC treatments for both species (pairwise comparisons in Table S10).

### 3.4 Egg and Sporophyte Production

**FIGURE 7:**
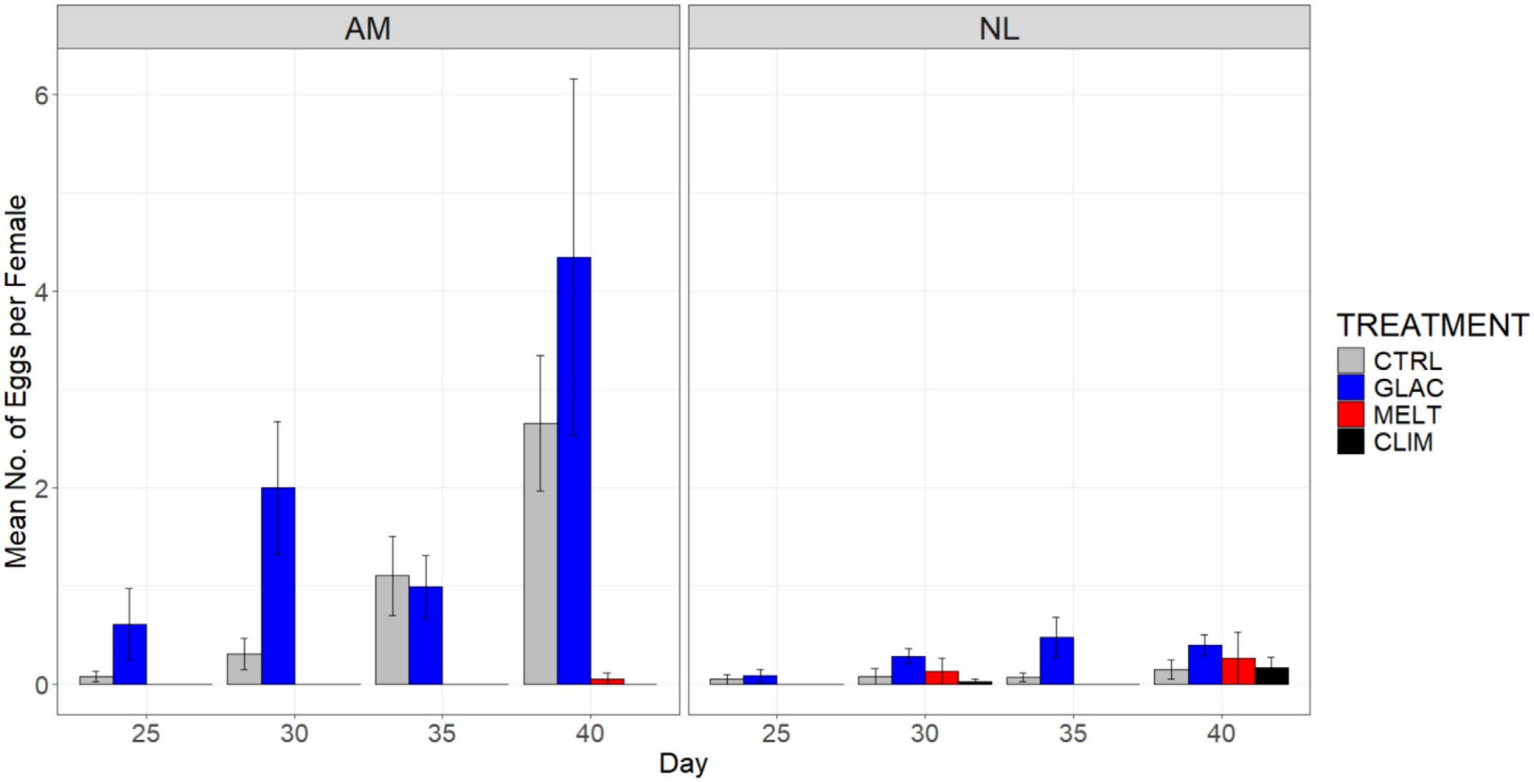
Mean number of eggs produced per female across stressor treatments for *Alaria marginata* (AM) and *Nereocystis luetkeana* (NL) specimens from 25 days up to 40 days. Error bars show standard error. Treatments are control (CTRL), climate change (CLIM), glacial runoff (GLAC) and meltwater (MELT), *n* = 5 petri dishes per treatment and species combination.

This study investigated female fecundity in terms of egg production and sporophyte production per female of each species. *A. marginata* females produced more eggs compared to *N. luetkeana* females, and overall we observed a three‐way Species × Treatment × Time interaction (*F*_7.85,83.76_ = 4.262, *p* < 0.05; Table S11). Upon splitting the dataset by species, we observed no effect of Treatment on egg production in *N. luetkeana*, but a significant Treatment × Time interaction for *A. marginata* (Table S12). Females of *A. marginata* produced more eggs in the CTRL and GLAC treatments compared to the CLIM and MELT treatments, where negligible counts of eggs were produced (Figure 7).

**FIGURE 8:**
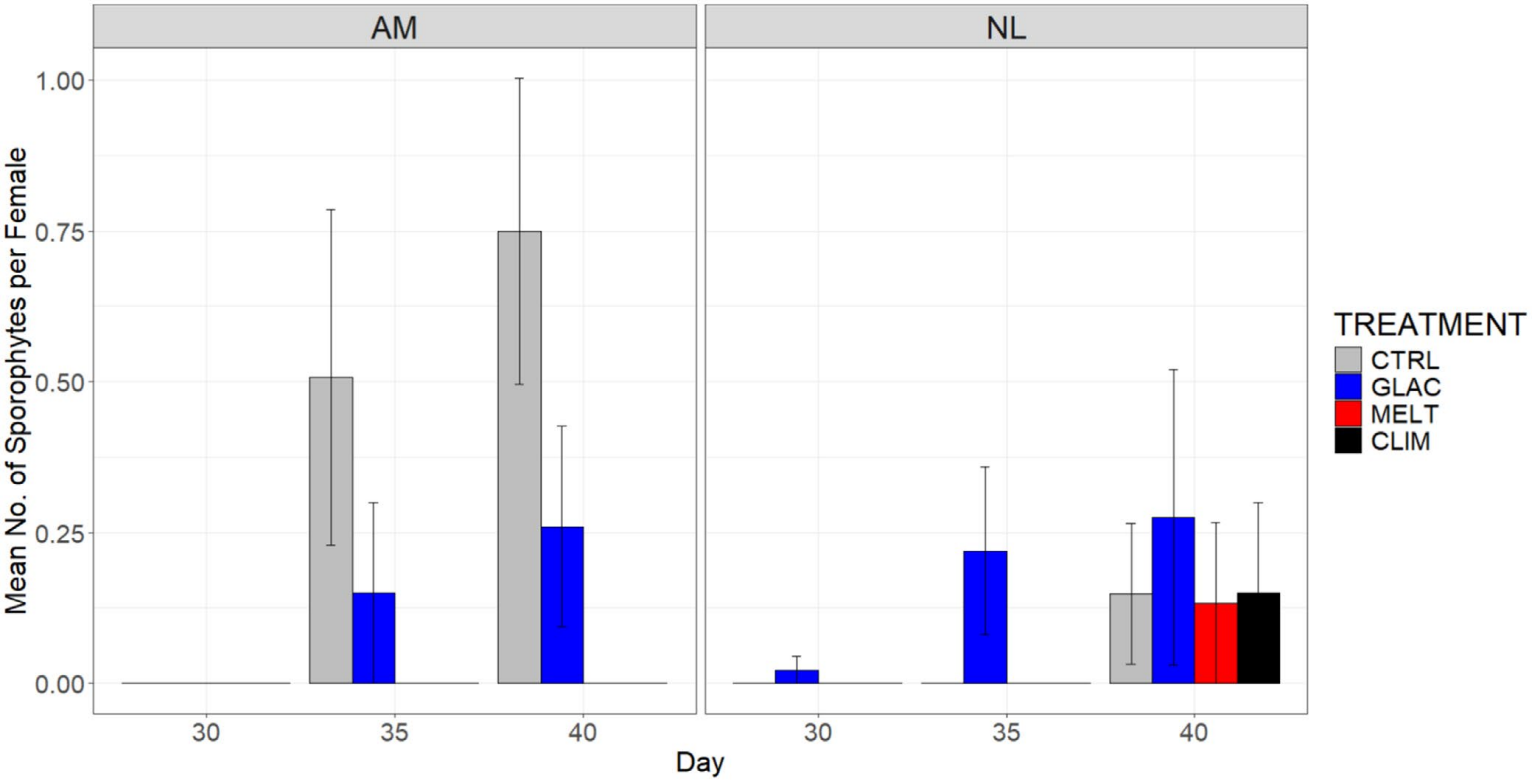
Mean number of sporophytes produced per female across stressor treatments for *Alaria marginata* (AM) and *Nereocystis luetkeana* (NL) specimens from 30 days up to 40 days. Error bars show standard error. Treatments are control (CTRL), climate change (CLIM), glacial runoff (GLAC) and meltwater (MELT), *n* = 5 petri dishes per treatment and species combination.

The development of sporophytes from eggs was analyzed with a three‐way Species × Treatment × Time interaction (*F*_15,160_ = 2.414, *p* < 0.05; Table S13). Upon splitting the dataset by species, we observed no effect of Treatment on sporophyte production in *N. luetkeana*, but a significant effect of Time—this species produced some sporophytes after 40 days of incubation regardless of stressor treatment (Figure 8). Once again, sporophyte production in *A. marginata* was determined by a two‐way Treatment × Time interaction (*F*_15,80_ = 4.380, *p* < 0.05; Table S14). Similar to egg production, females of *A. marginata* produced sporophytes in CTRL and GLAC treatments but not in CLIM and MELT treatments (Figure 8).

## 4 Discussion

This study investigated the impact of ecologically relevant combinations of climate‐related stressors on the early life stages of *Alaria marginata* and *Nereocystis luetkeana*. We observed evidence of stressor interactions including synergisms, wherein the effects of two or more stressors magnified each other to impact the species in question. Specifically, the CLIM scenario (T+, S−, D+), involving all three high stress conditions, had a markedly more severe effect on the life history responses measured here than either the GLAC (T, S−, D+) or MELT scenarios (T+, S−, D), both of which involve only two of three stressors at the high stress condition.

For both species considered here, the proportion of spores germinated was unaffected by treatment combination, although spores of *A. marginata* germinated more successfully than spores of *N. luetkeana* across all timepoints and treatments. This appears to contradict existing literature considering the effects of temperature (Farrugia Drakard et al. 2024), salinity (Farrugia Drakard et al. 2025), and sediment load (Picard et al. 2022) separately. However, this could be explained through an examination of the specific stressor levels utilized in these various studies. In terms of temperature, zoospores of *A. marginata* were found to germinate less successfully at 17°C than at 8°C (Farrugia Drakard et al. 2024). As the present study utilized 14°C as the high‐stress temperature condition, it is possible that zoospores of this species have a tolerance threshold somewhere between 14°C and 17°C. Indeed, Hoffman et al. (2003) report an upper survival threshold of 15°C for *A. marginata* zoospores. This may also be the case for salinity: Farrugia Drakard et al. (2024) found significant population‐level variation for both *N. luetkeana* and *A. marginata* in terms of the germination response to salinity. Specifically, spores of *A. marginata* from Juneau germinated more successfully at 32, 25, and 20 compared to 13. Once again, it is possible that this population exhibits a threshold tolerance to hyposaline conditions between 13 and 15, the high‐stress salinity condition utilized in this experiment. Finally, Picard et al. (2022) report a significant effect of suspended sediment load on spore performance in *Alaria esculenta* and *Saccharina latissima*. However, while the present study utilizes 0.1 g/L of sediment as the high‐stress condition, the cited study utilized 30 g/L (1.5 g per 50 mL) and 50 g/L (2.5 g per 50 mL) as sediment treatments. The stressor levels we have selected for this study are based on realistic ecological scenarios and climate projections, and therefore we can conclude that the zoospore stage of both *A. marginata* and *N. luetkeana* exhibits some resilience to climate stress and germination in these species is likely to be largely unaffected under future climate scenarios.

Conversely, the treatments utilized in this study had a significant impact on gametophyte density in both *A. marginata* and *N. luetkeana*. This appears to be driven mostly by the addition of sediment, as gametophyte density was lowest or very low for both species in the two treatments involving added sediment (CLIM and GLAC). Density in the MELT treatment was slightly higher for *N. luetkeana*—although not as high as in the CTRL (T, S, D) treatment—and markedly higher for *A. marginata* . This suggests that while hyposaline stress and high temperatures do have an effect on gametophyte density, their impact is not as severe as that of added sediment load. Increased sediment load is associated with physical scouring effects (Aumack et al. 2007; Picard et al. 2022; Zacher et al. 2016) and light attenuation (Livingston et al. 1998), both of which are likely to have an effect on the persistence of gametophytes on the benthos. Other studies have reported negative effects of sediment on spore attachment in *N. luetkeana, Eualaria fistulosa*, and *Laminaria solidungula* from sediment loads as low as 0.07 g/L (Deiman et al. 2012; Phelps et al. 2024). However, Phelps et al. (2024) report that sediment deposition did not have any effect on the ratio of gametophytes to settled spores, suggesting that sediment load does not impact spore viability once spores have attached. This implies that the effects on gametophyte density we report here may be a result of physical loss of spores through sediment scour rather than decreased spore viability or loss of the gametophytes themselves.

It is interesting that we observed a higher density of *A. marginata* gametophytes in the MELT treatment than in the CTRL treatment towards the end of the experiment. Gametophyte density for *A. marginata* in the MELT treatment was highly variable at these later timepoints (Day 25, Day 30) compared to earlier timepoints, suggesting that there is some degree of individual variation in how resilient gametophytes of this species are to thermal and hyposaline stress. *A. marginata* also exhibited a higher density of gametophytes in the MELT treatment compared to *N. luetkeana*. This is likely a result of the different ecological niches of these two species. While *N. luetkeana* is subtidal (Carney et al. 2005), *A. marginata* is intertidal (McConnico and Foster 2005). Therefore, gametophytes of *A. marginata* are exposed to a more variable thermal and salinity environment resulting from regular exposure to air, coastal precipitation runoff and their proximity to the surface freshwater layer.

Overall, *A. marginata* does appear to be more resilient to some degree of climate‐related stress, including the combinations of climate stressors utilized in this experiment. For example, with respect to gametophyte size, the mean for *A. marginata* was only slightly lower in the GLAC treatment than in the CTRL treatment at all timepoints, and some growth was still evident in the MELT treatment. Comparatively, gametophytes of *N. luetkeana* exhibited low mean gametophyte size in all treatment combinations other than the CTRL treatment. We can conclude that *A. marginata* gametophytes exhibit resilience to hyposaline stress in combination with sediment load (GLAC) and some resilience to thermal stress in combination with hyposaline stress (MELT). Once again, this is likely to be a result of environmental adaptation and corroborates results reported from previous studies (Farrugia Drakard et al. 2024, 2025). However, *A. marginata* and *N. luetkeana* gametophytes were equally impacted by the combination of all three high‐stress conditions in the CLIM treatment. This has significant implications for the survival of high‐latitude kelp populations in glaciated areas. It implies that under conditions of high combined climate stress, even populations and species that are adapted to either variable thermal stress, hyposaline conditions, or increased sediment loads may be unable to persist.

Approximately equal numbers of male and female gametophytes were observed for both species at all timepoints for every treatment combination. In certain cases, the number of females exceeded the number of males at later stages of the experiment. This result is likely due to how these gametophytes reproduce. Female gametophytes produce sessile eggs, while male gametophytes produce motile sperm. The sperm subsequently fertilize the eggs, which remain on the female gametophyte as they develop into sporophytes. Once males have released sperm, they degenerate and disappear, while the females persist to support the ongoing development of juvenile sporophytes (pers. obs.). Therefore, a higher number of females suggests successful production of gametes and fertilization.

The most striking trend we observed in this case is the high numbers of gametophytes which were unidentifiable as either male or female in the MELT and CLIM treatments. These are the only two treatment combinations in this study to involve a high temperature condition, and therefore this result confirms observations made in Farrugia Drakard et al. (2024)—namely that past a certain threshold, high temperatures are extremely detrimental to gametophyte development and reproduction. The cited study utilized a treatment of 17°C on *A. marginata*; we have now observed the same effect at 14°C on both *A. marginata* and *N. luetkeana*. Additionally, Weigel et al. (2023) showed that temperatures above 18°C were lethal to gametophytes of *N*. luetkeana from the Salish Sea (similarly high latitude), and no sporophytes were produced at these temperatures. This has significant implications for the persistence of wild populations of these species as global climate change progresses. Global temperatures are expected to rise between 0.8°C (RCP 2.6, very stringent emissions reductions) and 3.4°C (RCP 8.5, worst‐case scenario) by the year 2100 (van Vuuren et al. 2011; Davis et al. 2021). Under RCP 8.5, mean SST in summer in Juneau would rise to 15°C. Heatwave events are likely to exceed this approximation. Therefore, a temperature of 14°C is reasonable in terms of what gametophytes are likely to be exposed to towards the end of summer and in spring under future climate scenarios. We would strongly recommend that future studies investigate the impact of elevated temperatures on gametophytes of other high‐latitude kelp species (e.g., Hollarsmith et al. 2020; Mohring et al. 2014; Oppliger et al. 2012; Park et al. 2017). Our observations thus far have suggested that this phenomenon is broadly applicable to a number of species, but having tested only *N. luetkeana* and *A. marginata* we can only speculate.

Finally, there was no effect of treatment combination on the production of eggs and sporophytes in *N. luetkeana*, as gametophytes of this species produced very low numbers of eggs overall and, consequently, low numbers of sporophytes. Conversely, *A. marginata* appeared to be sensitive to treatment in this regard. Gametophytes of this species produced no eggs and no sporophytes in the CLIM and MELT treatments but were able to produce eggs and sporophytes in the CTRL and GLAC treatments. Once again, it is notable that the CLIM and MELT treatments both involve a high temperature condition, and therefore it is likely that the lack of successful reproduction in these treatments is a consequence of temperature impacts on the sexual development of the gametophytes, as discussed in the previous paragraph. This appears to confirm that the apparent deformation of gametophytes as a result of high temperatures does indeed lead to reproductive consequences. These deformed gametophytes appear to be unable to produce eggs and therefore do not produce sporophytes. This has implications for the viability of populations, as thermal stress events that impact the gametophyte stage may result in failure to produce eggs and sporophytes across a population, leading to mass mortality events.

There is a general lack of information regarding the responses of high‐latitude kelp species to climate‐related environmental stress. The results presented here show that both *N. luetkeana* (a subtidal canopy‐former) and *A. marginata* (an intertidal subcanopy species) are sensitive to combinations of thermal, hyposaline, and sediment stress. This may have an impact on the development of gametophytes and successful reproduction in these species and may therefore have implications for the ongoing persistence of wild kelp populations in future ocean conditions.

## Author Contributions

**Veronica Farrugia Drakard:** conceptualization (lead), data curation (lead), formal analysis (lead), funding acquisition (equal), investigation (lead), methodology (lead), visualization (lead), writing – original draft (lead), writing – review and editing (lead). **Jordan A. Hollarsmith:** conceptualization (supporting), funding acquisition (supporting), project administration (equal), resources (equal), supervision (equal), writing – review and editing (supporting). **Michael S. Stekoll:** conceptualization (supporting), funding acquisition (supporting), project administration (equal), resources (equal), supervision (equal), writing – review and editing (supporting).

## Conflicts of Interest

The authors declare no conflicts of interest.

## Supporting information


Appendix S1.

Appendix S2.

## Data Availability

All raw data have been provided along with this manuscript as Appendix S1 and S2.
