# Supplementary material for: Impact of Multiple Climate Stressors on Early Life Stages of North Pacific Kelp Species
Source: Ecol Evol. 2025 Jun 20;15(6):e71661. doi: 10.1002/ece3.71661 (PMC12181651; doi:10.1002/ece3.71661)
Supplement: Supplementary file 1 — Appendix S1. [file ECE3-15-e71661-s001.docx]

Supplementary Material

1. **Zoospore survival and germination**

Table 1: Sphericity-corrected three-way mixed ANOVA for arcsine-transformed proportions of germinated zoospores to total zoospore count in A. marginata and N.luetkeana, across treatments and species with Time as a within-group variable. Bold text indicates a significant effect – main effects are disregarded if included in a significant higher-level interaction.

| Effect | *DF_n_* | *DF_d_* | *F* | *p* | *ges* |
| --- | --- | --- | --- | --- | --- |
| Species | 1 | 32 | 6.411 | **0.016** | 0.059 |
| Treatment | 3 | 32 | 2.468 | 0.080 | 0.068 |
| Time | 1 | 32 | 4.516 | **0.041** | 0.088 |
| Species*Treatment | 3 | 32 | 1.562 | 0.218 | 0.044 |
| Species*Time | 1 | 32 | 9.318 | **0.005** | 0.166 |
| Treatment*Time | 3 | 32 | 0.295 | 0.829 | 0.019 |
| Species*Treatment*Time | 3 | 32 | 0.231 | 0.874 | 0.015 |

1. **Gametophyte abundance**

Table 2: Sphericity-corrected three-way mixed ANOVA for log-transformed gametophyte abundance in A. marginata and N.luetkeana, across treatments and species with Time as a within-group variable. Bold text indicates a significant effect – main effects are disregarded if included in a significant higher-level interaction.

| Effect | *DF_n_* | *DF_d_* | *F* | *p* | *ges* |
| --- | --- | --- | --- | --- | --- |
| Species | 1 | 31 | 0.591 | 0.448 | 0.009 |
| Treatment | 3 | 31 | 16.852 | **1.12e^-6^** | 0.436 |
| Time | 1.59 | 49.27 | 4.254 | **0.027** | 0.067 |
| Species*Treatment | 3 | 31 | 5.321 | **0.004** | 0.196 |
| Species*Time | 1.59 | 49.27 | 1.679 | 0.201 | 0.102 |
| Treatment*Time | 4.77 | 49.27 | 2.226 | 0.069 | 0.102 |
| Species*Treatment*Time | 4.77 | 49.27 | 1.077 | 0.384 | 0.052 |

Table 3: Sphericity-corrected one-way ANOVAs for log-transformed gametophyte abundance, showing effect of stressor treatment in each species and effect of species for each stressor treatment. Bold text indicates a significant effect – main effects are disregarded if included in a significant higher-level interaction.

| Effect | *DF_n_* | *DF_d_* | *F* | *p* | *ges* |
| --- | --- | --- | --- | --- | --- |
| **TREATMENT** |  |  |  |  |  |
| *Alaria marginata* (AM) | 3 | 75 | 13.4 | **8.92e^-7^** | 0.348 |
| *Nereocystis luetkeana* (NL) | 3 | 76 | 41.2 | **1.32e^-15^** | 0.619 |
| **SPECIES** |  |  |  |  |  |
| Climate change (CLIM) | 1 | 37 | 0.607 | 1 | 0.016 |
| Control (CTRL) | 1 | 38 | 6.70 | 0.06 | 0.15 |
| Glacial melt (GLAC) | 1 | 38 | 3.14 | 0.34 | 0.076 |
| Meltwater (MELT) | 1 | 38 | 13.7 | **0.003** | 0.266 |

Table 4: Significant pairwise t-test comparisons with Bonferroni correction between stressor treatments for gametophyte abundance in A. marginata and N. luetkeana.

| Species | Comparison | *p* |
| --- | --- | --- |
| *Alaria marginata* | CTRL v GLAC | 0.002 |
|  | CLIM v MELT | 1.06e^-4^ |
|  | GLAC v MELT | 1.18e^-6^ |
| *Nereocystis luetkeana* | CLIM v CTRL | 1.43e-^14^ |
|  | CTRL v GLAC | 2.59e^-13^ |
|  | CLIM v MELT | 0.026 |
|  | CTRL v MELT | 5.95e^-9^ |

1. **Gametophyte size and sex ratios**

Table 5: Sphericity-corrected three-way mixed ANOVA for square-root transformed mean gametophyte size in A. marginata and N. luetkeana, across stressor treatments and species with Time as a within-group variable. Bold text indicates a significant effect – main effects are disregarded if included in a significant higher-level interaction.

| Effect | *DF_n_* | *DF_d_* | *F* | *p* | *ges* |
| --- | --- | --- | --- | --- | --- |
| Species | 1 | 32 | 14.003 | **7.19e^-4^** | 0.182 |
| Treatment | 3 | 32 | 50.033 | **3.49e^-12^** | 0.705 |
| Time | 2.96 | 94.68 | 55.005 | **1.59e^-20^** | 0.458 |
| Species*Treatment | 3 | 32 | 9.116 | **1.65e^-4^** | 0.303 |
| Species*Time | 2.96 | 94.68 | 5.803 | **0.001** | 0.082 |
| Treatment*Time | 8.88 | 94.68 | 2.112 | **0.037** | 0.089 |
| Species*Treatment*Time | 8.88 | 94.68 | 1.336 | 0.230 | 0.058 |

Table 6: Sphericity-corrected one-way ANOVAs for square-root transformed mean gametophyte size, showing effect of stressor treatment in each species and effect of species for each stressor treatment. Bold text indicates a significant effect – main effects are disregarded if included in a significant higher-level interaction.

| Effect | *DF_n_* | *DF_d_* | *F* | *p* | *ges* |
| --- | --- | --- | --- | --- | --- |
| **TREATMENT** |  |  |  |  |  |
| *Alaria marginata* (AM) | 3 | 116 | 58.9 | **6.6e^-23^** | 0.604 |
| *Nereocystis luetkeana* (NL) | 3 | 116 | 42.1 | **3.52e^-18^** | 0.521 |
| **SPECIES** |  |  |  |  |  |
| Climate change (CLIM) | 1 | 58 | 4.51 | 0.152 | 0.072 |
| Control (CTRL) | 1 | 58 | 2.88 | 0.38 | 0.047 |
| Glacial melt (GLAC) | 1 | 58 | 45.1 | **3.52e^-8^** | 0.437 |
| Meltwater (MELT) | 1 | 58 | 21.3 | **8.84e^-5^** | 0.269 |

Table 7: Significant pairwise t-test comparisons with Bonferroni correction between stressor treatments for gametophyte size in A. marginata and N. luetkeana.

| Species | Comparison | *p* |
| --- | --- | --- |
| *Alaria marginata* | CLIM v CTRL | 9.36e^-13^ |
|  | CLIM v GLAC | 5.05e^-12^ |
|  | CLIM v MELT | 6.9e^-7^ |
|  | CTRL v GLAC | 6.6e^-4^ |
|  | CTRL v MELT | 1.27e^-12^ |
|  | GLAC v MELT | 2.86e^-10^ |
| *Nereocystis luetkeana* | CLIM v CTRL | 9.72e^-12^ |
|  | CTRL v GLAC | 3.76e^-9^ |
|  | CTRL v MELT | 4.92e^-15^ |


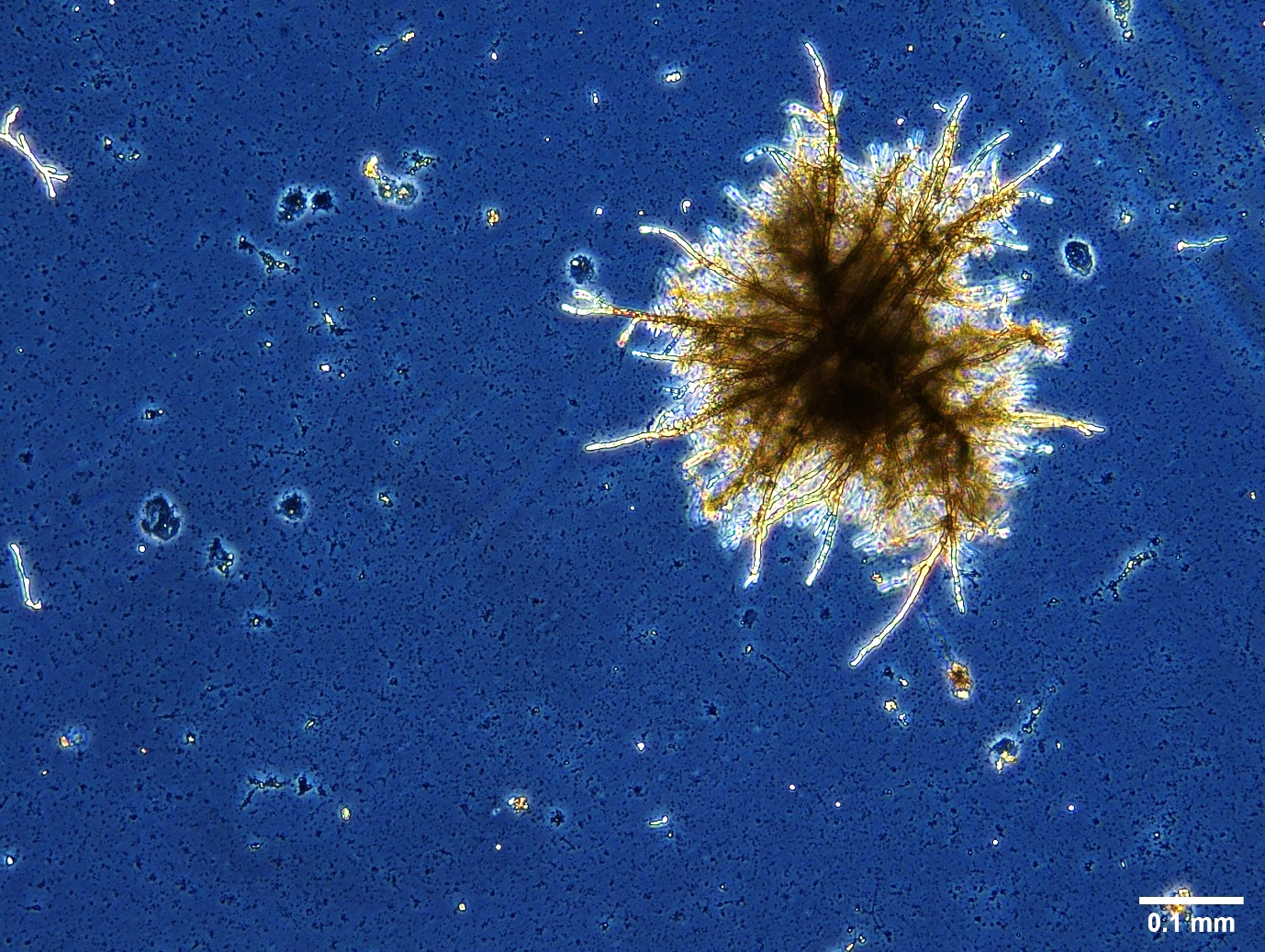

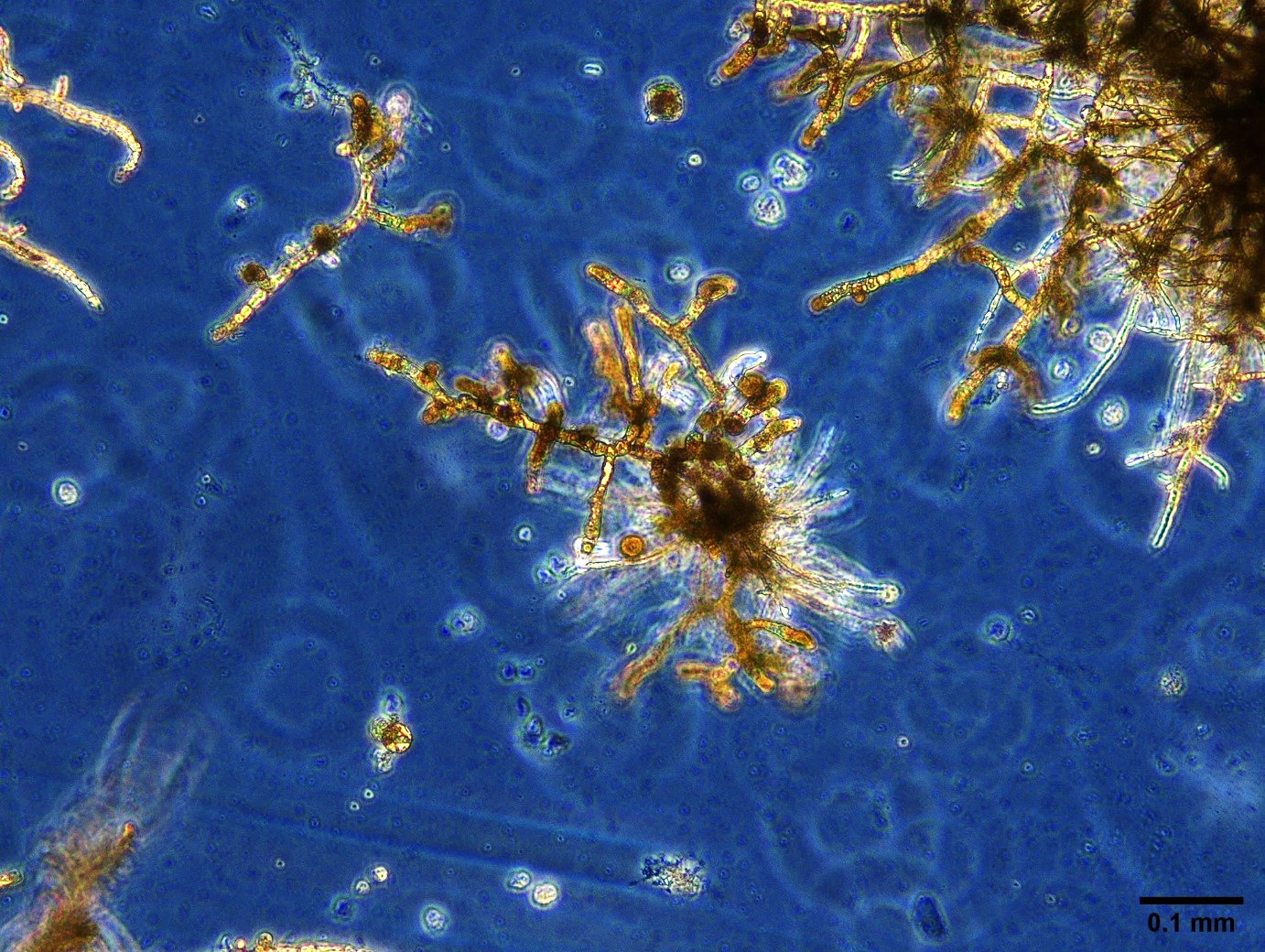

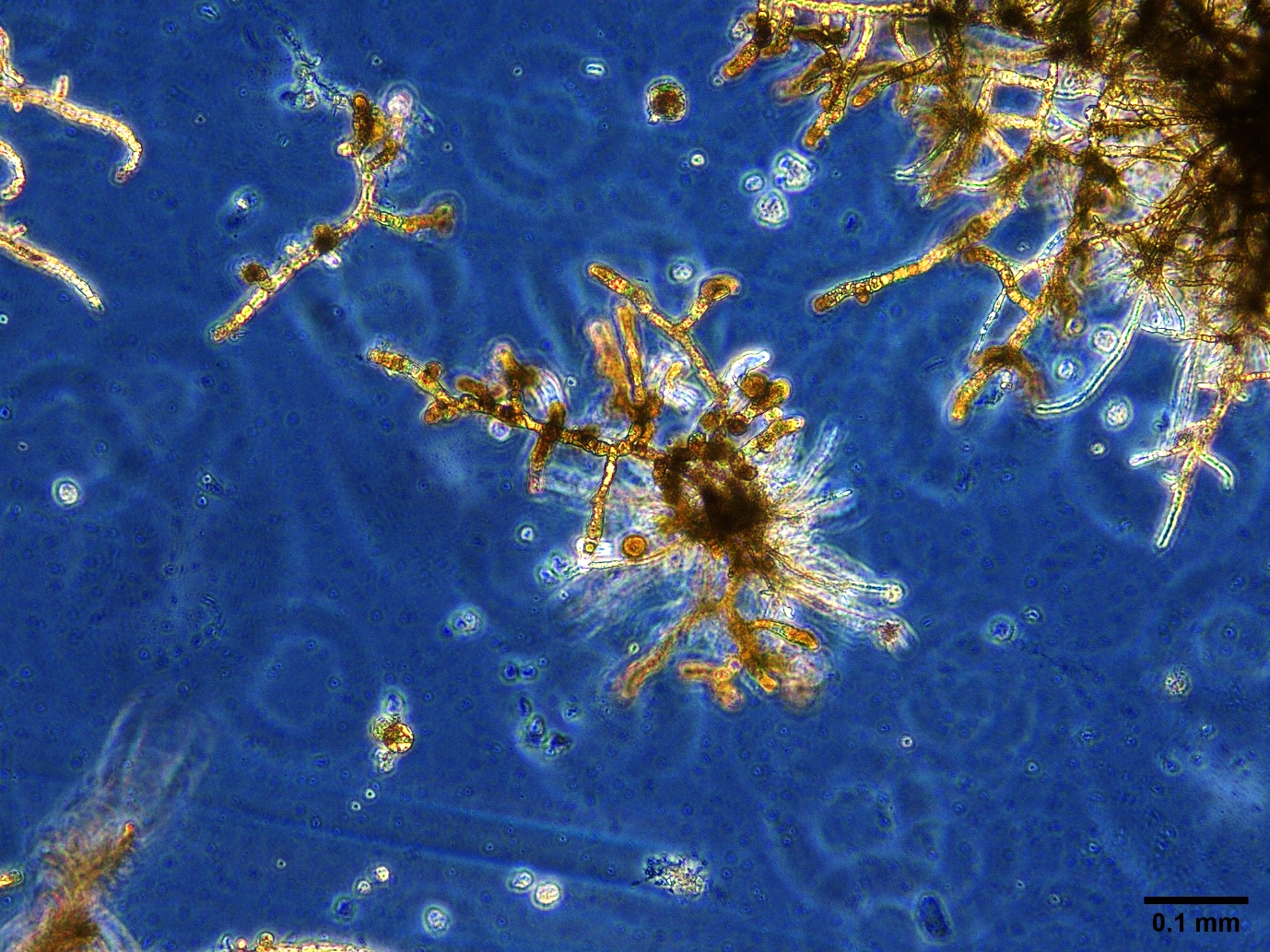

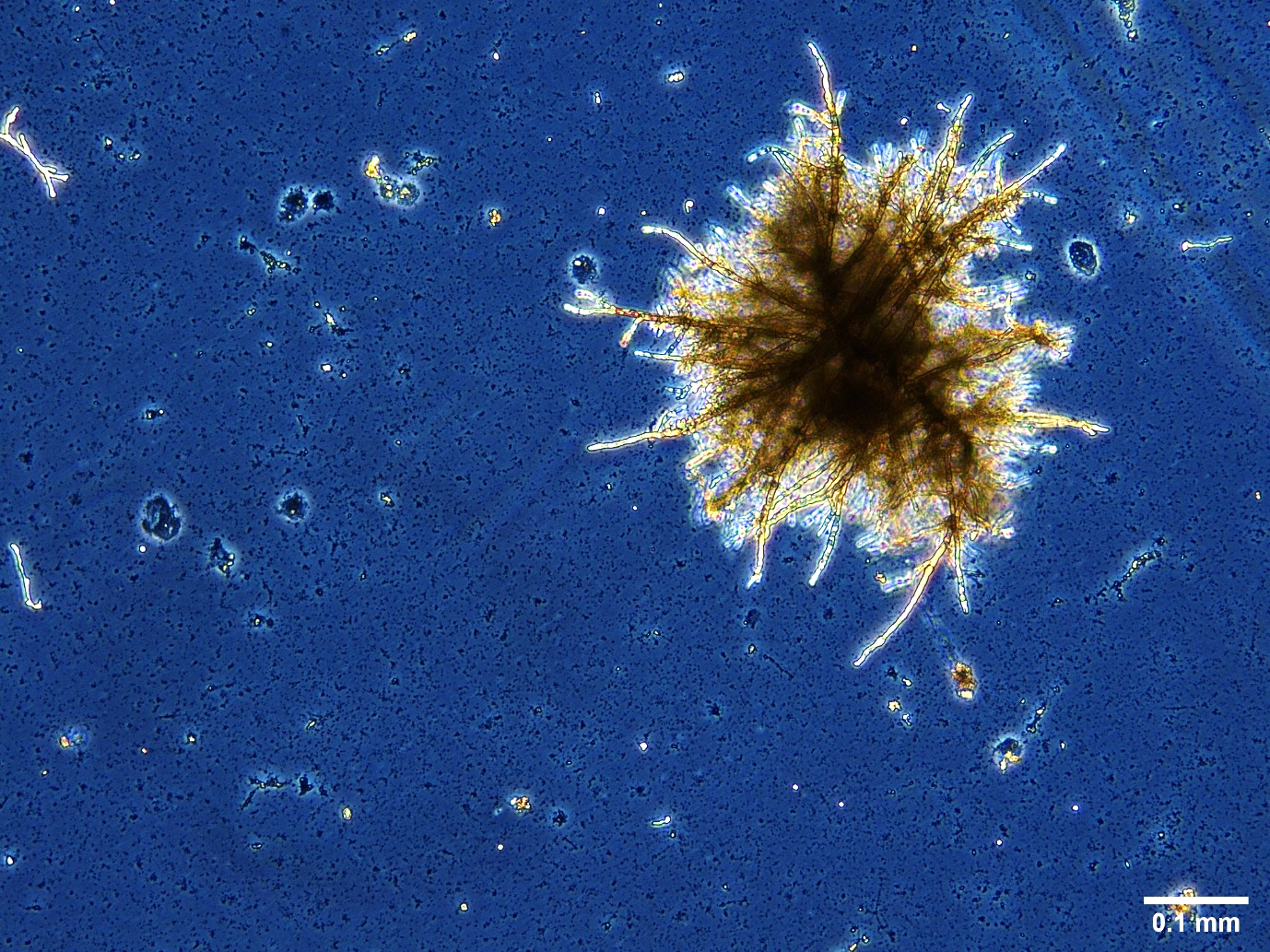


**A**

**B**


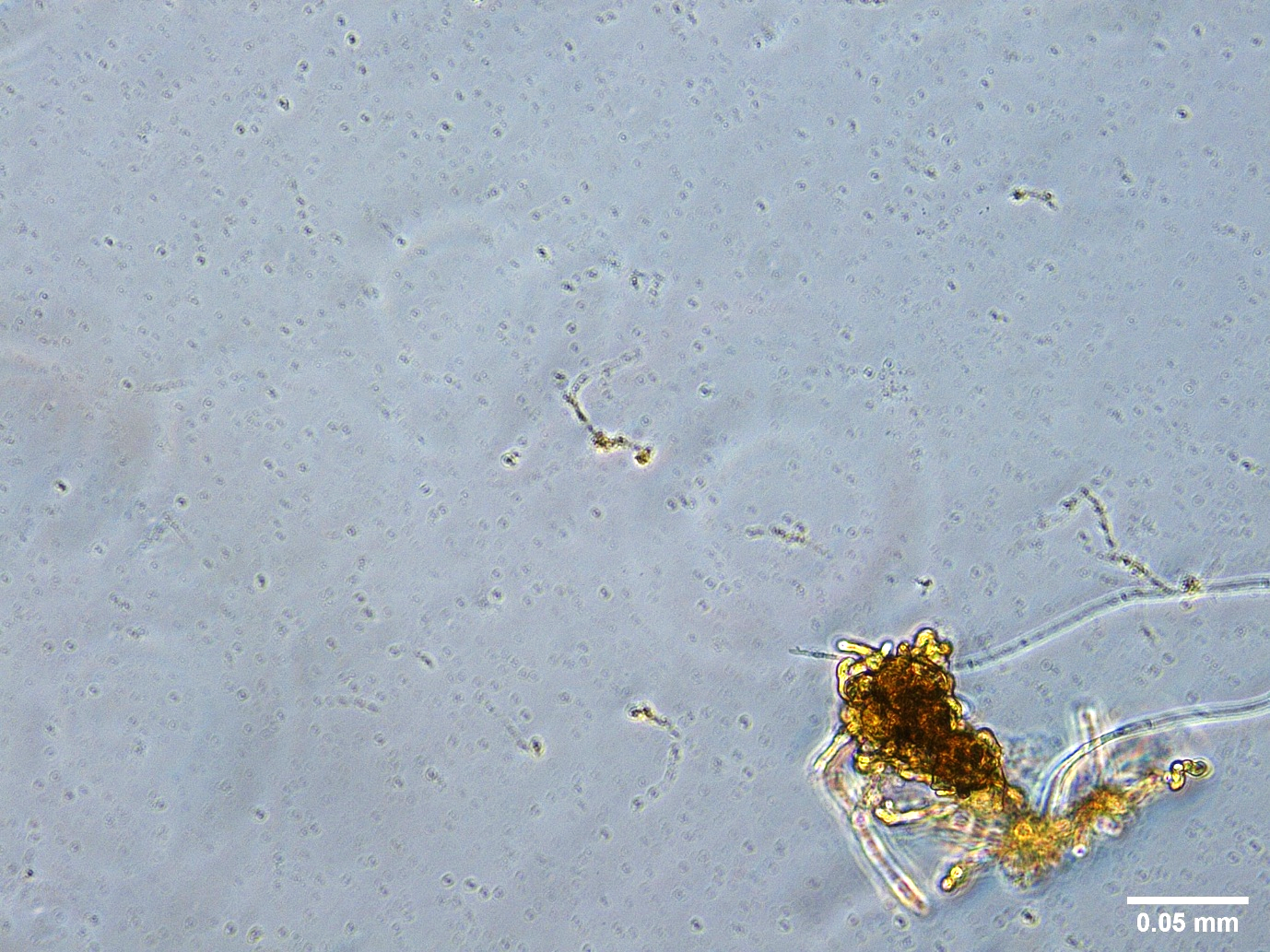


**C**

Figure 1: Gametophytes of Alaria marginata after 35 days of growth. A: healthy female in control conditions; B: healthy male in control conditions; C: deformed gametophyte in the MELT (meltwater) treatment – high temperature, low salinity, normal suspended sediment load.


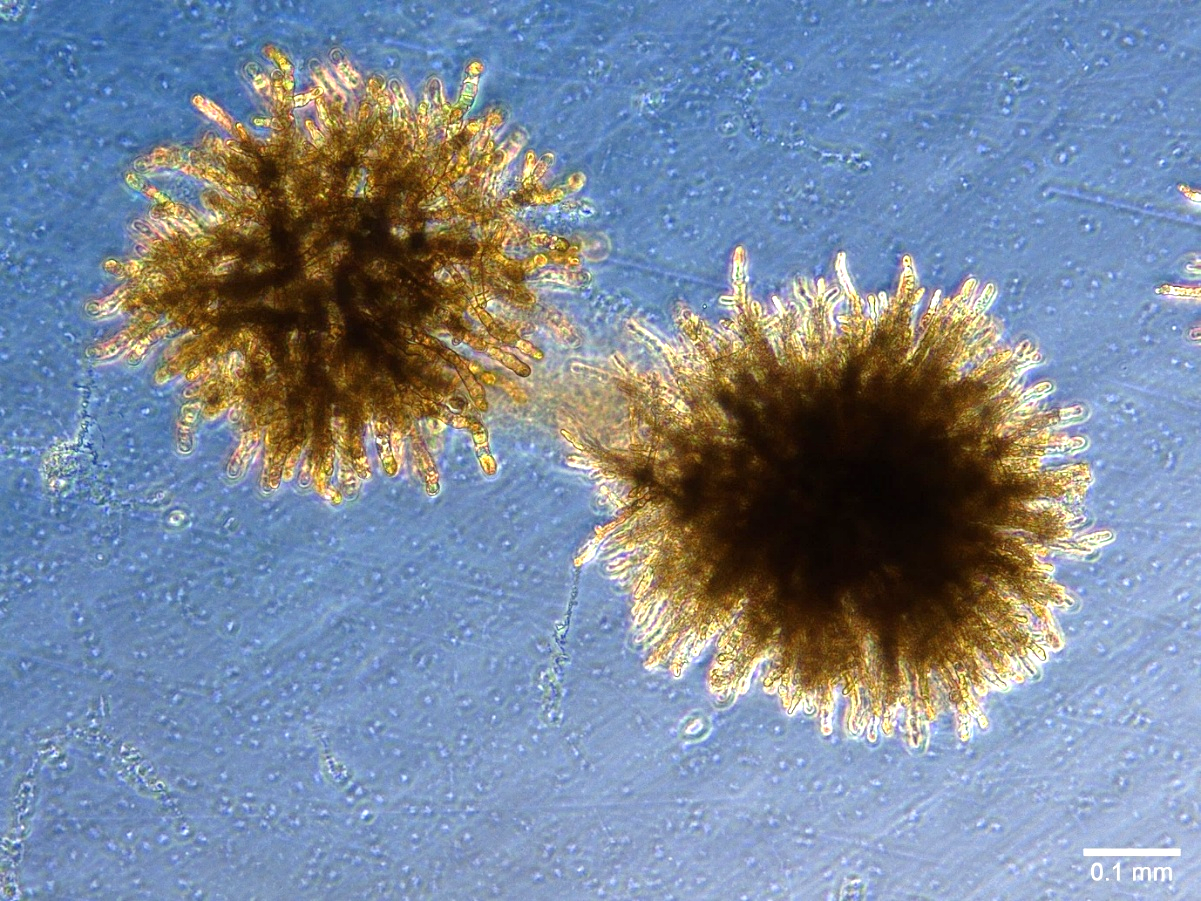


**A**


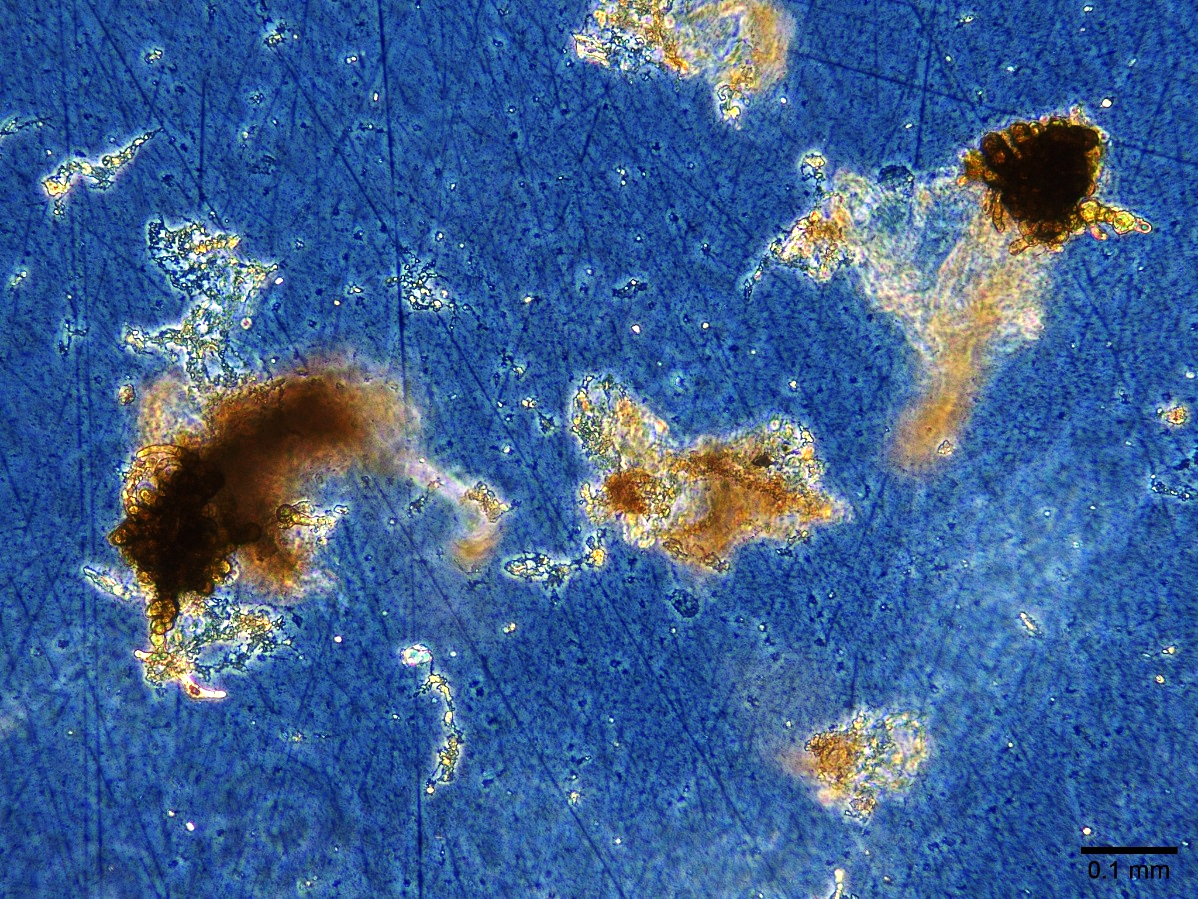


**B**

Figure 2: Gametophytes of Nereocystis luetkeana after 35 days of growth. A: healthy female (left) and male (right) in control conditions; B: deformed gametophytes in the MELT (meltwater) treatment – high temperature, low salinity, normal suspended sediment load.

Table 8: Sphericity-corrected three-way mixed ANOVA for square-root transformed number of gametophytes of unidentifiable sex in A. marginata and N. luetkeana, across stressor treatments and species with Time as a within-group variable. Bold text indicates a significant effect – main effects are disregarded if included in a significant higher-level interaction.

| Effect | *DF_n_* | *DF_d_* | *F* | *p* | *ges* |
| --- | --- | --- | --- | --- | --- |
| Species | 1 | 32 | 3.540 | 0.069 | 0.037 |
| Treatment | 3 | 32 | 82.687 | **3.68e^-15^** | 0.730 |
| Time | 3.21 | 102.86 | 18.459 | **4.6e^-10^** | 0.273 |
| Species*Treatment | 3 | 32 | 4.007 | **0.016** | 0.116 |
| Species*Time | 3.21 | 102.86 | 0.933 | 0.433 | 0.019 |
| Treatment*Time | 9.64 | 102.86 | 7.910 | **3.85e^-9^** | 0.326 |
| Species*Treatment*Time | 9.64 | 102.86 | 3.386 | **8.6e^-4^** | 0.171 |

Table 9: Sphericity-corrected two-way ANOVAs for square-root transformed number of gametophytes of unidentifiable sex in A. marginata and N. luetkeana, across stressor treatments with Time as a within-group variable. Bold text indicates a significant effect – main effects are disregarded if included in a significant higher-level interaction.

| Effect | *DF_n_* | *DF_d_* | *F* | *p* | *ges* |
| --- | --- | --- | --- | --- | --- |
| **A. MARGINATA** |  |  |  |  |  |
| Time | 2.65 | 42.34 | 6.036 | **0.002** | 0.228 |
| Treatment | 3 | 16 | 59.547 | **6.6e^-9^** | 0.706 |
| Treatment*Time | 7.94 | 42.34 | 5.141 | **1.69e^-4^** | 0.431 |
| **N. LUETKEANA** |  |  |  |  |  |
| Time | 2.63 | 42.08 | 16.397 | **8.62e^-7^** | 0.337 |
| Treatment | 3 | 16 | 35.321 | **2.75e^-7^** | 0.769 |
| Treatment*Time | 7.89 | 42.08 | 6.577 | **1.62e^-5^** | 0.380 |

Table 10: Significant pairwise t-test comparisons with Bonferroni correction between stressor treatments for number of unidentifiable gametophytes in A. marginata and N. luetkeana.

| Species | Comparison | *p* |
| --- | --- | --- |
| *Alaria marginata* | CLIM v MELT | 3.44e^-8^ |
|  | CTRL v MELT | 4.76e-^15^ |
|  | GLAC v MELT | 3.38e^-11^ |
| *Nereocystis luetkeana* | CLIM v CTRL | 8.82e^-9^ |
|  | CLIM v GLAC | 0.038 |
|  | CLIM v MELT | 1.23e^-5^ |
|  | CTRL v GLAC | 2.71e^-4^ |
|  | CTRL v MELT | 2.33e^-28^ |
|  | GLAC v MELT | 3.63e^-8^ |

1. **Egg and sporophyte production**

Table 11: Sphericity-corrected three-way mixed ANOVA for square-root transformed number of eggs produced per female in A. marginata and N. luetkeana, across stressor treatments and species with Time as a within-group variable. Bold text indicates a significant effect – main effects are disregarded if included in a significant higher-level interaction.

| Effect | *DF_n_* | *DF_d_* | *F* | *p* | *ges* |
| --- | --- | --- | --- | --- | --- |
| Species | 1 | 32 | 19.386 | **1.12e^-4^** | 0.135 |
| Treatment | 3 | 32 | 27.828 | **4.8e^-9^** | 0.402 |
| Time | 2.62 | 83.76 | 27.854 | **1.15e^-11^** | 0.393 |
| Species*Treatment | 3 | 32 | 10.177 | **7.36e^-5^** | 0.197 |
| Species*Time | 2.62 | 83.76 | 5.871 | **0.002** | 0.120 |
| Treatment*Time | 7.85 | 83.76 | 7.219 | **3.6e^-7^** | 0.334 |
| Species*Treatment*Time | 7.85 | 83.76 | 4.262 | **2.72e^-4^** | 0.229 |

Table 12: Sphericity-corrected two-way ANOVAs for square-root transformed number of eggs produced per female in A. marginata and N. luetkeana, across stressor treatments with Time as a within-group variable. Bold text indicates a significant effect – main effects are disregarded if included in a significant higher-level interaction.

| Effect | *DF_n_* | *DF_d_* | *F* | *p* | *ges* |
| --- | --- | --- | --- | --- | --- |
| **A. MARGINATA** |  |  |  |  |  |
| Time | 2.07 | 33.16 | 20.894 | **1.07e^-6^** | 0.508 |
| Treatment | 3 | 16 | 32.946 | **4.44e^-7^** | 0.562 |
| Treatment*Time | 6.22 | 33.16 | 7.575 | **3.10e^-5^** | 0.529 |
| **N. LUETKEANA** |  |  |  |  |  |
| Time | 2.65 | 42.48 | 7.174 | **8.26e^-4^** | 0.224 |
| Treatment | 3 | 16 | 2.983 | 0.062 | 0.166 |
| Treatment*Time | 7.96 | 42.48 | 1.329 | 0.256 | 0.139 |

Table 13: Sphericity-corrected three-way mixed ANOVA for square-root transformed number of sporophytes produced per female in A. marginata and N. luetkeana, across stressor treatments and species with Time as a within-group variable. Bold text indicates a significant effect – main effects are disregarded if included in a significant higher-level interaction.

| Effect | *DF_n_* | *DF_d_* | *F* | *p* | *ges* |
| --- | --- | --- | --- | --- | --- |
| Species | 1 | 32 | 0.764 | 0.388 | 0.005 |
| Treatment | 3 | 32 | 5.027 | **0.006** | 0.093 |
| Time | 5 | 160 | 14.208 | **1.68e^-11^** | 0.258 |
| Species*Treatment | 3 | 32 | 4.046 | **0.002** | 0.076 |
| Species*Time | 5 | 160 | 0.619 | 0.686 | 0.015 |
| Treatment*Time | 15 | 160 | 2.974 | **3.32e^-4^** | 0.179 |
| Species*Treatment*Time | 15 | 160 | 2.414 | **0.003** | 0.150 |

Table 14: Sphericity-corrected two-way ANOVAs for square-root transformed number of sporophytes produced per female in A. marginata and N. luetkeana, across stressor treatments with Time as a within-group variable. Bold text indicates a significant effect – main effects are disregarded if included in a significant higher-level interaction.

| Effect | *DF_n_* | *DF_d_* | *F* | *p* | *ges* |
| --- | --- | --- | --- | --- | --- |
| **A. MARGINATA** |  |  |  |  |  |
| Time | 5 | 80 | 9.356 | **4.77e^-7^** | 0.332 |
| Treatment | 3 | 16 | 11.422 | **2.98e^-4^** | 0.245 |
| Treatment*Time | 15 | 80 | 4.380 | **6.58e^-6^** | 0.411 |
| **N. LUETKEANA** |  |  |  |  |  |
| Time | 5 | 80 | 0.988 | 0.424 | 0.049 |
| Treatment | 3 | 16 | 5.215 | **3.41e^-4^** | 0.190 |
| Treatment*Time | 15 | 80 | 0.786 | 0.689 | 0.096 |
